# Use of Air-Classification Technology to Manage Mycotoxin and Arsenic Contaminations in Durum Wheat-Derived Products

**DOI:** 10.3390/foods11030304

**Published:** 2022-01-24

**Authors:** Alessandro Cammerata, Rosita Marabottini, Viviana Del Frate, Enrica Allevato, Samuela Palombieri, Francesco Sestili, Silvia Rita Stazi

**Affiliations:** 1 Council for Agricultural Research and Economics, Research Centre for Engineering and Agro-Food Processing, Via Manziana 30, 00189 Rome, Italy; alessandro.cammerata@crea.gov.it (A.C.); viviana.delfrate@crea.gov.it (V.D.F.); 2 Department for Innovation in Biological, Agro-Food and Forest Systems (DIBAF), University of Tuscia, Via San Camillo de Lellis snc, 01100 Viterbo, Italy; marabottini@unitus.it; 3 Department of Environmental and Prevention Sciences (DiSAP), University of Ferrara, Via Luigi Borsari 46, 44121 Ferrara, Italy; enrica.allevato@unife.it; 4 Department of Agriculture and Forest Sciences (DAFNE), University of Tuscia, Via San Camillo de Lellis snc, 01100 Viterbo, Italy; palombieri@unitus.it; 5 Department of Chemical, Pharmaceutical and Agricultural Sciences (DOCPAS), University of Ferrara, Via Luigi Borsari 46, 44121 Ferrara, Italy

**Keywords:** durum wheat, air classification, inorganic contaminants, organic contaminants, arsenic, mycotoxins

## Abstract

Mycotoxins are the most common natural contaminants and include different types of organic compounds, such as deoxynivalenol (DON) and T-2 and HT-2 toxins. The major toxic inorganic elements include those commonly known as heavy metals, such as cadmium, nickel, and lead, and other minerals such as arsenic. In this study, micronisation and air classification technologies were applied to durum wheat (*Triticum turgidum* ssp. *durum* L.) samples to mitigate inorganic (arsenic) and organic contaminants in unrefined milling fractions and final products (pasta). The results showed the suitability of milling plants, providing less refined milling products for lowering amounts of mycotoxins (DON and the sum of T-2 and HT-2 toxins) and toxic inorganic elements (As, Cd, Ni, and Pb). The results showed an As content (in end products) similar to that obtained using semolina as raw material. In samples showing high organic contamination, the contamination rate detected in the more bran-enriched fractions ranged from 74% to 150% (DON) and from 119% to 151% (sum of T2 and HT-2 toxins) as compared to the micronised samples. Therefore, this technology may be useful for manufacturing unrefined products with reduced levels of organic and inorganic contaminants, minimising the health risk to consumers.

## 1. Introduction

Currently, cereal grains are the primary source of carbohydrates in human nutrition worldwide. The Food and Agriculture Organization (FAO) forecasts that world grain utilisation in 2021/2022 will reach a record level of 2809 million tons. The FAO’s forecast for total wheat utilisation is 777 million tons, which is 2.4% (18.5 million tons) higher than that in 2020/2021 [1]. Durum wheat is a crop of primary importance, as semolina (obtained by milling durum wheat grain) is the basic raw material for the realisation of several highly consumed end products (pasta, couscous, bulgur, local breads) and is mainly used as a traditional food for Mediterranean populations, and it is highly appreciated worldwide.

Cereals such as wheat provide most of the energy in diets, and their consumption in the form of unrefined end products provides health advantages because they are rich in bioactive compounds. Among these, antioxidant molecules are of great interest because they are involved in lowering the risk of cardiovascular diseases, cancers, gastrointestinal disorders, and type 2 diabetes [2,3,4,5,6].

In general, wholemeal products are realised by mixing suitable amounts of bran, germ, and endosperm fractions so that they are as similar as possible to the natural grains [7]. However, the consumption of bran-enriched products could increase the risk of consumers’ exposure to food-borne organic and inorganic contaminants, the main ones being mycotoxins, toxic elements such as arsenic, and heavy metals. All of these compounds can persist from the raw matter to the end products, in addition to so-called processing compounds, such as acrylamide [8]. The environment plays a key role as the main source of contamination in grains, such as wheat, starting from the cultivation field and ending in storage. Even though minerals are ubiquitous in the Earth’s crust, anthropogenic activities are increasingly resulting in the contamination of water and soil with toxic metals and metalloids [9,10,11]. Among toxic minerals, arsenic (As), cadmium (Cd), and lead (Pb) are ranked as the most hazardous substances owing to their toxicity, prevalence, and potential for human exposure [12]. Among the metals of great concern, cadmium (Cd) is uniformly distributed within the endosperm, whereas lead (Pb) and nickel (Ni) are mainly located in the outer teguments [13,14,15]. European legislation has established maximum tolerable levels of several metals and metalloids in foodstuffs. However, the maximum levels of Pb, Cd, and tin (Sn) are currently established and monitored in cereals, whereas the maximum arsenic content has been established only for rice (0.200 µg/g) [16,17].

Mycotoxins, which are produced by several fungi growing on cereal grains, are of great concern, as they add organic contaminants to a wide range of food commodities [17,18]. Deoxynivalenol (DON) and T-2/HT-2 toxins belong to type B and A, respectively, groups of chemically related compounds named trichothecenes. They are the most widespread *Fusarium* toxins in small grain cereals, including durum wheat (*Triticum turgidum* ssp. *durum* L.), which constitutes the main source through which humans and animals are exposed to these types of mycotoxins [19,20]. These mycotoxins are produced by fungi that colonise the kernel, starting in its outer layers, where they mostly accumulate. Since the coating structures end up in the bran milling fraction, bran-enriched foods represent an increased risk of human exposure to mycotoxins [21,22,23,24,25].

The maximum tolerable limits for DON in food products have been set by the European Commission [17]. However, to date, there are no legal limits for T-2 and HT-2 toxins in food and feed. Nevertheless, the European Commission recommendation on the content of T-2 and HT-2 in cereals and cereal indicates 100 μg/kg for the sum of T-2 and HT-2 as the maximum level in cereal grains [26].

The use of new technologies, such as debranning, micronisation, and air classification, at the starting phase of wheat processing is effective in reducing the presence of undesirable compounds within the milling products [27,28,29]. In fact, micronisation combined with air classification provides suitable solutions to obtain less refined products with improved safety and nutritional aspects, ensuring optimal technological quality. Recently, a study of the process carried out using updated pilot plants allowed the selection of several unrefined milling fractions obtained from micronised and air-classified durum wheat samples. Therefore, three types of fractions were selected based on the results obtained in previous assays [30,31]. These fractions proved to be of interest owing to the yield percentage, particle size composition, and health benefits associated with a higher content of bran (fibre, antioxidants, minerals, etc.).

The goal of this study was to assess the reliability of recently updated milling plants (microniser and air classifier) to minimise the presence of DON, T-2 and HT-2 toxins, and toxic elements (mainly arsenic) along the production chain.

## 2. Materials and Methods

### 2.1. Durum Wheat Samples

**Figure 1:**
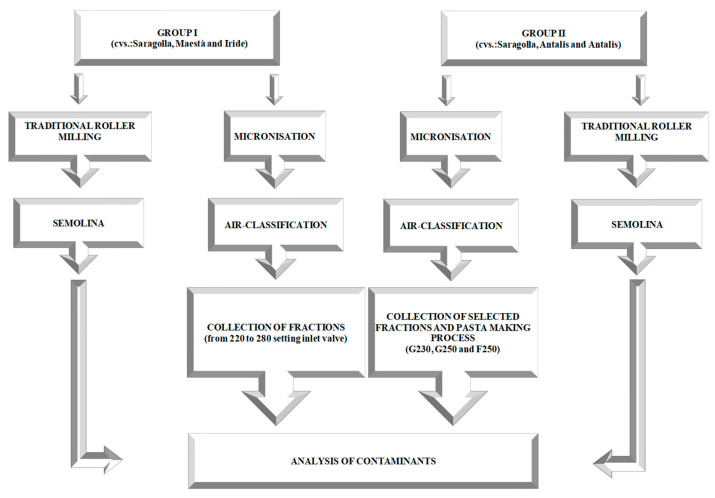
Flow chart of the experimental plan.

A process study was carried out on three durum wheat grain samples (group I) of cultivars Saragolla, Maestà, and Iride. All of them were grown in conventional farming fields in Apulia (Southern Italy) during the 2018–2019 season (Figure 1). The use of these three samples allowed the identification of air-classifying conditions that were suitable for the aim of this study. In particular, group I samples were selected from those collected within the “AsFRUM” research project. The criteria adopted in the choice of samples concerned both the type and the rate of contamination. Moreover, the use of three different cultivars only served to achieve greater variability in the grain characteristics without accounting for the cultivar as a factor. On the basis of the results obtained in the process study, three additional durum wheat grain samples (group II) were used only under the selected air-classifying conditions previously identified in the process study. These group II samples were collected within the aforesaid research project during the same cropping year (2018–2019) in conventional growing fields located in three regions of the centre-south of Italy. More specifically, one durum wheat sample (Saragolla) was grown in the Lazio region (Central Italy), and the other two samples from the cv. Antalis were grown in the Marche region (Central Italy) and in the Basilicata region (Southern Italy) (Figure 1). In this case (group II), the selection criteria adopted for grain samples also concerned both the type and the rate of contamination. Three analytical replicates were carried out for all of the materials tested, as detailed below.

### 2.2. Micronisation and Air Classification Processes

The milling plants (microniser and air classifier) used in this study were already supplied to our laboratories and recently updated [30].

Briefly, innovative advances in the microniser pilot plant (model 32,300, KMXi-300-7,5; Separ Microsystem S.a.s, Brescia, Italy) included the introduction of a hammer crusher impeller, which reduced the internal cross-section and a sieving grid (Ø = 0.7 mm), which produced a more homogeneous milled product than the traditional type. The air-classifier pilot plant (model SX-LAB; Separ Microsystem S.a.s, Brescia, Italy) designed for a sample particle size up to a set limit (Ø ≤ 1.5 mm) was improved through the insertion of a programmable logic controller (PLC) capable of managing the airflow inside the separation chamber. Moreover, the orbits located in the inner part of the chamber were improved by designing a progressively decreasing inside diameter. Each micronised sample (groups I and II) was split into 1.5 kg aliquots. The micronisation step did not require preventive conditioning of the grains. The samples included in group I were subjected to the air classification process by setting the airflow inlet valve from 220 to 280 as opening conditions for each cycle at a time. At the end of each cycle, fractions of types G (heavier coarse particles) and F (fine particles) were collected. The samples of group II were only air-classified at the 230 and 250 settings based on a previous evaluation (see Figure 1). Therefore, the three air-classified fractions (F250, G250, and G230) were collected and used in the subsequent steps.

### 2.3. Traditional Roller Milling Process

The traditional roller milling process was carried out as a reference process. Grain samples were conditioned by adding tap water until a moisture value of 17% was achieved, and the samples were left to rest for 24 h. This treatment facilitated the processes of both stripping the kernels and softening the endosperm, making grain samples suitable for processing in the traditional roller milling plant (Bühler, model MLU 202, Uzwil, Switzerland). The main milling products were collected: semolina refined through a sieving treatment (sieve types: 38GG, 40GG, and 44GG) using a suitable pilot plant sieving system (NAMAD Impianti, Rome, Italy), bran fractions (coarse and refined types), and fine middlings.

### 2.4. Pasta-Making Process

A pilot press plant (NAMAD Impianti, Rome, Italy) was used, and the “spaghetti” format (Ø = 1.6 mm) was adopted in the pasta-making process. The pasta samples were dried for 18 h at low temperature (50 °C) using a suitable dryer plan (AFREM, Lyon, France).

### 2.5. Mycotoxin Analysis

Sampling was carried out based on representative criteria, and all milled samples (from micronisation, air classification, and traditional roller milling) were stored at 2–8 °C until analysis [32]. DON content was assessed using the Ridascreen^®^ DON kit (Ridascreen^®^ DON method, R-Biopharm AG, Darmstadt, Germany) following the manufacturer’s instructions. Specifically, extraction at a ratio of 1 part sample to 5 parts deionised water was performed, and then the sample was shaken vigorously for 3 min. The extract was filtered through a Whatman no. 1 filter, and the filtrate was collected as a sample. The limit of detection (LOD) of the enzyme-linked immunosorbent assay (ELISA) was 18.5 μg/kg. The recovery range obtained with this method was 85–110% of the sample. Deionised water was obtained from the Water Purification System Zeener Power I (Human Corporation, Seoul, Korea). The basic robotic immunoassay operator (BRIO, SEAC, Radim Group, Florence, Italy) was employed, and the absorbance data were acquired using a Sirio-S microplate reader (SEAC, Radim Group, Florence, Italy). The ELISA method for the DON content in durum wheat samples had already been validated by comparison with chromatographic (HPLC) analysis [33]. The sum of T-2 and HT-2 toxins was measured by ELISA analysis (Veratox^®^ T2/HT2 toxin, Neogen, Lansing, MI, USA) according to the manufacturer’s instructions. Extraction at a ratio of 1 part sample to 5 parts 70% methanol was carried out, and then the sample was shaken vigorously for 3 min. The extract was filtered through a Whatman no. 1 filter, and the filtrate was collected as a sample. The limit of quantification (LOQ) was 25 µg/kg. The ELISA method for measuring the sum of T-2 and HT-2 toxin content in durum wheat samples was assessed by comparison with ultra-performance liquid chromatography (UPLC) analysis [34]. Absorbance values were read using the Sirio-S microplate reader (SEAC, Radim Group, Florence, Italy), and the RIDA^®^ Soft Win software (R-Biopharm AG, Darmstadt, Germany) was used for the quantification of mycotoxins in samples.

### 2.6. Toxic Elements

Micronised samples and all air-classified G and F fractions were processed to determine the concentrations of As, Cd, Pb, and Ni. Each sample underwent an initial step of acid digestion using a laboratory high-pressure microwave oven (Mars plus CEM, Cologno al Serio (BG), Italy) with a power of 1.200 W. Approximately 200 mg of the dry sample was placed in contact with 7.5 mL of 67% *v*/*v* HNO_3_ for one hour, and then 0.5 mL of HCl was added and left to digest for one hour; finally, 2 mL of H_2_O_2_ was added and left to digest for 30 min. At the end of the predigestion process, the obtained acid solution was mineralised inside a microwave, with a heating program in which a temperature of 180 °C was reached in 37 min and incubation at 180 °C continued for 15 min. At the end of the digestion procedure, the samples were appropriately diluted with high-purity water (18 MΩ/cm) obtained from a Milli-Q water purification system (Millipore, Bedford, MA, USA) and then filtered (DISMIC 25HP PTFE syringe filter, pore size = 0.45 mm, Toyo Roshi Kaisha, Ltd., Tokyo, Japan) and stored in a plastic screw-cap tube (Nalgene, New York, NY, USA). Each experiment was performed in triplicate.

Elemental quantification was performed using an inductively coupled plasma optical emission spectrometer (ICP-OES) with an axial configuration (8000 DV, Perkin Elmer Inc. Waltham, MA, USA) equipped with an ultrasonic nebuliser (Teledyne Cetac Tecnologies, Omaha, USA). To assess the concentration levels of trace elements, calibration standards were prepared and treated in the same way as the samples before dilution (multi-element standard solution, CaPurAn, CPAchem, Stara Zagora, Bulgaria). The frequencies used for the determinations were: Cd 228.8 nm, Fe 259.9 nm, Cu 324.7 nm, Pb 220.3, Ni 231.6 nm, and As 197.69 nm.

The European Reference Material ERM-BC21 was used as the standard material to assess the accuracy of the measurements.

The super-pure-grade reagents used for microwave-assisted digestion were as follows: hydrochloric acid (36% HCl), nitric acid (69% HNO_3_), and hydrogen peroxide (30% H_2_O_2_); highly pure water (18 MΩ/cm) was used to dilute the standards and prepare the samples throughout the chemical procedure.

### 2.7. Statistical Analysis

The results for the mycotoxin content were subjected to analysis of variance (ANOVA and Tukey test) using the statistical software PAST v. 2.12, Oslo, Norway [35].

## 3. Results

### 3.1. Determination of Mycotoxin Concentration

**Figure 2:**
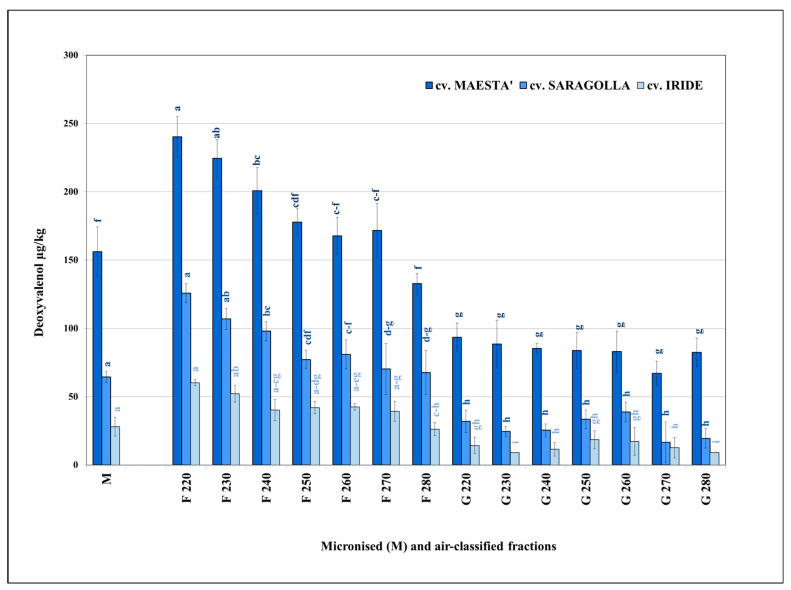
Average content of DON in the micronised (M) and air-classified fractions (F and G) of the durum wheat samples belonging to group I; different letters within each cultivar indicate significant differences (*p* < 0.05), *n* = 3.

All air-classified G and F fractions (220–280) collected from group I samples were analysed to show the distribution of DON and T-2 and HT-2 toxins (sum of the two types) within the same samples. The results clearly highlight the influence of the process conditions on the DON content in the milling fractions collected (Figure 2).

The average levels of DON In micronised samples ranged between 28 µg/kg (cv. Iride) and 156 µg/kg (cv. Maestà). The highest mycotoxin content was detected in the F fractions, with a clear decreasing trend from fraction F220 to fraction F280 in all samples tested. From F220 to F240, which had the highest degree of contamination, the differences were statistically significant (*p* < 0.05) as compared with the corresponding micronised samples. A marked reduction in mycotoxin content was also found in all G fractions, with no significant differences (*p* > 0.05) between the most contaminated cultivar samples (Saragolla and Maestà). In this regard, all G fractions were significantly different (*p* < 0.05) from the F fractions.

The aforementioned general trend was even more marked in the case of the residual content, expressed as a percentage, compared to the micronised samples, all of which were set equal to 100%. In fact, the contamination rate varied from a minimum of 155% (cv. Maestà) to a maximum of 215% (cv. Iride) in the F220 fractions, whereas it decreased in the F280 fractions to values even lower than those of the micronised samples (cv. Maestà = 86% and cv. Iride = 94%, both in F280). Among the G fractions, the maximum rate achieved was 66% (G250) as compared to the micronised sample (cv. Iride).

**Figure 3:**
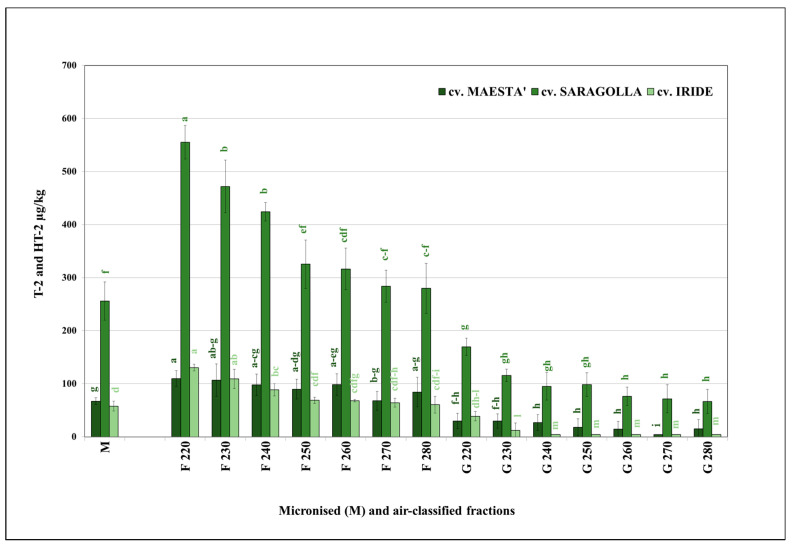
Average content of the sum of T-2 and HT-2 toxins in the micronised (M) and air-classified fractions (F and G) of the durum wheat samples belonging to group I; different letters within each cultivar indicate significant differences (*p* < 0.05), *n* = 3.

A trend similar to DON was also found for the sum of T-2 and HT-2 toxin content, though to a greater extent (Figure 3). In fact, the F fractions F220, F230, and F240 showed significantly higher levels of contamination (*p* < 0.05) with a clear further reduction in the remaining F fractions as compared to the average values of the corresponding micronised samples, ranging from 58 µg/kg (cv. Iride) to 256 µg/kg (cv. Saragolla). A marked reduction in mycotoxin content was detected in all G fractions without significant differences (*p* > 0.05) in any samples between fractions G240 and G280, but they were significantly different (*p* < 0.05) from the F fractions. Moreover, similarly to DON, a marked effect of the air classification process was evident in the percentage evaluation as compared to micronised samples (set equal to 100%) In fact, within all F220 fractions, the percentage rates varied from a minimum of 164% (cv. Maestà) to a maximum of 225% (cv. Iride), with a tendency to decrease as the opening of the airflow inlet valve increased until reaching levels close to those of the micronised samples (Maestà = 105% in F270). A further decreasing trend was observed in G fractions, which did not exceed the percentage value of 46% for G230 (cv. Maestà), except for G220 in all samples (maximum value = 67% for cv. Iride).

**Figure 4:**
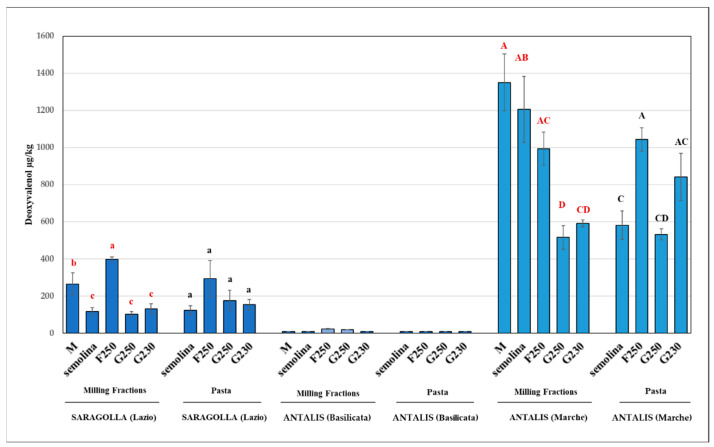
Group II: average content of DON in the micronised (M) and air-classified fractions (F and G) of the durum wheat samples that tested positive; different letters within each group indicate significant difference (*p* < 0.05). Different colored letters are referred to different sample types (red for milling fractions and black for pasta), *n* = 3.

The results for the DON contents only in the selected fractions (group II) showed a trend similar to that of group I (Figure 4). In fact, in the case of the more contaminated sample (cv. Antalis, Marche region) showing the highest DON content (1350 µg/kg) in the micronised sample, there was no significant difference (*p* > 0.05) between micronised and F250 samples, and significant differences (*p* < 0.05) between micronised and G fractions were confirmed. The pasta-making process effect tended to limit the concentration of DON in the F fractions as compared to the micronised sample, and only the F250 fraction (1043 µg/kg) was significantly different (*p* < 0.05) from semolina in Antalis (Marche).

**Figure 5:**
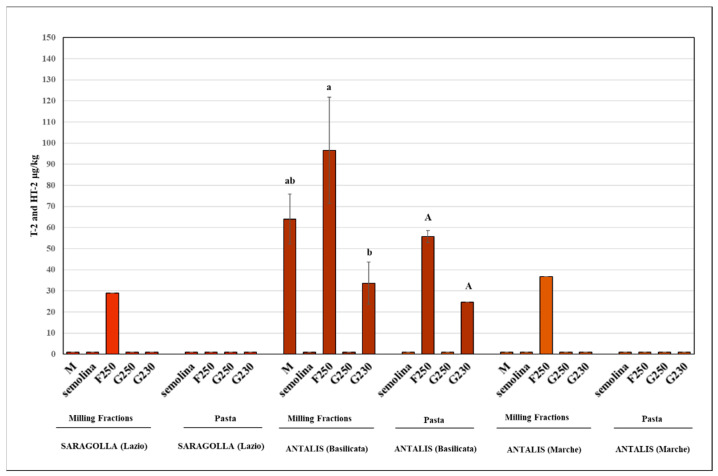
Group II: average values of the sum of the T-2 and HT-2 toxins in the micronised (M) and in the air-classified fractions (F and G) of the durum wheat samples that tested positive; different letters within each group indicate significant difference (*p* < 0.05), *n* = 3; data without a letter indicate single values (F250).

As regards contamination by T-2 and HT-2 toxins, which is defined as the sum of the two, in the most contaminated sample (cv. Antalis, Basilicata), there was a trend showing the highest contamination rate in the bran-enriched F250 fraction (97 µg/kg), but the difference was not significant (*p* > 0.05) compared to the micronised sample (64 µg/kg) (Figure 5). Conversely, a marked decrease from 34 µg/kg in the G230 fraction to undetectable levels (less than the limit of quantification (<LOQ)) in the G250 fraction was observed. Moreover, in micronised samples that did not show detectable levels of the sum of T-2 and HT-2 toxins (cv. Saragolla, Lazio region, and cv. Antalis, Marche region), the “latent” presence of toxins, just below the LOQ (25 µg/kg), led to their detection only in the F250 fraction (maximum value: 37 µg/kg). The absence of contamination was confirmed in all G250 fractions analysed, whereas a marked effect of the pasta-making process on the most contaminated fractions (F250) was observed.

### 3.2. Determination of Toxic Elements

All air-classified G and F fractions (220–280) collected from the group I samples were analysed to determine the distribution of As, Cd, Ni, and Pb (Table 1 and Table 2).

As in the case of As and Cd, a more marked reduction in the Ni content of the G fractions than that in the F fractions was observed. In particular, F220 and F230 (cv. Maestà and cv. Saragolla, respectively) showed a trend toward lower values of Ni content than the corresponding micronised samples, but the difference was significant (*p* < 0.05) only for the cv. Saragolla samples. This trend was not confirmed in cv. Iride samples because all F fractions showed values higher than the micronised fractions, with relevance for F260 (0.031 µg/g). In addition, this fraction type tended toward higher levels of Cd in all cultivar samples tested. The G fractions had a lower rate of contamination than the corresponding micronised samples, especially with regard to G280, which reached a maximum value of 0.005 µg/g (cv. Saragolla). Regarding the cv. Iride G fractions, only G250 (0.035 µg/g) and G260 (0.004 µg/g) had detectable Ni content, which was undetectable for the micronised and the remaining G samples.

Higher Pb content in micronised samples from cv. Maestà (0.157 µg/g) and cv. Saragolla (0.152 µg/g) compared to cv. Iride (0.022 µg/g) was detected. An appreciable trend toward a reducing rate due to the technological process of milling fractions was confirmed in all cultivar samples, especially for G fractions from G250 to G280. Although not always significant, a general tendency toward values lower than those of the micronised samples was observed from F230 to F250 in all cultivar samples. However, in the case of cv. Iride, only the F230 value (0.016 µg/g) was significantly lower than that of the micronised sample. Table 3 shows the results for As, Cd, Ni, and Pb for group II. The As contamination was significantly decreased in all fractions as compared to the corresponding micronised samples. More specifically, the F250 and G250 fractions maintained the lowest rate of contamination (maximum percentage residue: 52% in F250, cv. Saragolla), which is even lower than that of semolina.

Regarding Cd contamination, the presence of the element was unchanged compared to micronised samples. Undetectable Ni content was observed in all micronised and milled samples, and the same results were obtained for all fraction types tested.

Regarding Pb contamination, the fractions F250 and G250 had undetectable levels of this element (content < LOD) in micronised samples. Moreover, the F250 and G250 fractions showed a significantly (*p* < 0.05) lower rate of contamination than the micronised sample (0.138 µg/g) from cv. Antalis (Basilicata).

Through the pasta-making process, a slight decrease in the content of As in semolina was achieved, which proved to not be significantly (*p* > 0.05) different from the air-classified fractions (Table 4).

## 4. Discussion

This study aimed to address the issue of organic and inorganic contamination in durum wheat-derived food products, which has already been preliminarily examined [29]. In the present assay, updated micronising and air classification plants were used as reliable tools to improve the quality characteristics of products. Moreover, the choice of the three fractions assayed in the second series of samples (group II) also took into account the previous assessment of the main quality characteristics (i.e., milling yield, particle size composition, and ash content). These characteristics shown by all air-classified fractions (from the 220 to 280 setting) were previously assayed in a process study [30].

As a whole, the three air-classified fractions assayed in this study were the best compromise between the presence of bran particles and several contaminants in the products.

Moreover, the use of several cultivars showing different grain characteristics was deemed suitable only to introduce more variability within the durum wheat samples tested.

The results obtained show a clear effect of the process on the mycotoxin contamination of both milling and final products while retaining a non-negligible amount of bran particles in the air-classified mixes. Both types of mycotoxins were concentrated in the F250 fraction, with a clear trend toward a strong reduction in the G fractions. However, different behaviours of DON and T-2/HT-2 toxins were observed (Figure 4 and Figure 5). In fact, the only semolina sample that was significantly different (*p* < 0.05) from both micronised and F250 fractions was the sample with slight DON contamination (cv. Saragolla, region Lazio), whereas in the case of high contamination (cv. Antalis, Marche region), no significant difference (*p* > 0.05) was detected for these fractions. These results confirm the high capacity of DON-producing fungi to penetrate the inner parts of the kernel, thus colonising the endosperm structure with negative effects on semolina, as previously shown [36]. Moreover, the pasta-making process did not produce significant differences (*p* > 0.05) among the semolina and G fractions, probably due to the high content of semolina particles in the G fractions [30].

In the case of a higher rate of contamination of the sum of T-2 and HT-2 toxins (cv. Antalis, Basilicata region), a strong mycotoxin accumulation in F250 (151%) and a decreased amount in G230 (53%) were noted as compared to the micronised sample. Mycotoxin contamination persisted in these air-classified fractions (F250 and G230), albeit to a lesser extent (88% and 39%, respectively) in the end product (pasta). Conversely, the presence of toxins was not detected in semolina. This result is consistent with previous studies showing the greater difficulty of managing toxin-producing fungi (i.e., *F. langsethiae*) colonising the inner endosperm of the kernel and thus strongly limiting semolina contamination [21,22,23,24,25,26,27,28,29,30,31,32,33,34,35,36,37]. Interestingly, the toxin content (sum of T-2 and HT-2) tended to concentrate in the F250 fraction, and a higher bran content was clearly demonstrated by the measurement that was close to the limit of quantification of the ELISA test (25 µg/g). This limit must be defined as the “zone” with the greatest uncertainty of quantification. However, this was not observed in the corresponding pasta samples analysed.

As a whole, the results for both types of mycotoxins underline the importance of the starting levels of grain contamination for determining the rate of contamination in both the air-classified fractions and final product (pasta). For this reason, the air classification technology, when intended as a management tool for mycotoxin contamination in products, must be used only for grain samples with absent or slight contamination below the mandatory limits [16,17,18,19,20,21,22,23,24,25,26]. In fact, only in this manner can the residual contamination rate in milling and final products be maintained below the established safe levels.

The data from the detection of inorganic contaminants showed an important effect of the technological process on the content of toxic elements in the different fractions, which is in agreement with previous assays [14,15,38]. In particular, the latter assays highlighted the effect of processing under controlled pilot plant conditions on the levels of As, Cd, Pb, and Ni, mainly by producing a marked trend toward a reduction in As content in the milling fractions. This latter aspect was confirmed in our study for pasta manufactured both with semolina and with the use of each of the three selected fractions (F250, G250, and G230).

The residual content of elements assayed in the fractions decreased as compared to the micronised samples, except for several fractions in which the opposite trend was observed. Moreover, considering the lower starting contamination rates of micronised samples with Cd, Ni, and Pb as compared to As, only the latter element was analysed in the end product (pasta) as the inorganic contaminant of major interest for the purposes of this work.

From the results obtained, we cannot select a fraction where the average content of all toxic elements analysed was consistently significantly lower than that of the micronised sample. However, as shown by the first series tested (group I), a significant trend of the G fractions toward a reduction in inorganic element contents, as compared to the F fractions, was clearly observed. This could be due to the higher content of bran particles in F fractions than in G fractions, which conversely contained higher amounts of semolina residues. Furthermore, the latter effect was evident as the progressive airflow opening increased. In addition, based on the contamination rates observed, the three air-classified fractions (F250, G250, and G230), previously selected based on qualitative evaluation, were confirmed as suitable products for the pasta-making process [30,31].

The results for the second series of samples (group II) proved to be of particular interest because it seemed to confirm the process effect shown in the first series (group I), mainly regarding As contamination. In fact, in all samples, the lowest As content was found in the G250 fraction, with decreasing rates ranging from 75% (cv. Saragolla, Lazio region) to 86% (cv. Antalis, Basilicata region). The presence of Cd and Pb was reduced, and thus, lower levels were present in the chosen fractions than in the semolina samples.

The assessment of As content in the end product was of great interest due to its role as an important source of consumers’ direct exposure to food-borne contaminants [11]. The clear trend toward lower rates of As contamination shown by the three selected fractions (F250, G250, and G230) can provide greater safety in manufacturing unrefined end products. In fact, the same three air-classified fractions showed no significant difference as compared to semolina, even in the fraction with a higher content of bran residue (F250). The lower As content as a process effect in pasta manufacturing is in agreement with a previous assay [15].

## 5. Conclusions

This work addressed the management of several organic (mycotoxins) and inorganic (mainly arsenic) contaminants in the manufacturing of unrefined durum wheat products. The importance of reducing the risk of consumers’ exposure to toxic compounds derives from the role of pasta as an appreciated and widely consumed food worldwide. The results obtained confirmed the reliability of the upgraded technological process to provide air-classified milling fractions characterised by the strong containment of several contaminants, and thus, the process is suitable for manufacturing high-quality, unrefined end products. Therefore, from a technological point of view, suitable management protocols and desirable results were achieved for preparing the main product of durum wheat (pasta), with a focus on ensuring consumers’ safety.

## Figures and Tables

**Table 1:** Group I: average values ± standard error (SE) of arsenic (As), cadmium (Cd), nickel (Ni), and lead (Pb) contents in the micronised samples and F-type air-classified fractions; d.m.: dry matter; LOD: limit of detection. Different letters indicate significant difference (*p* < 0.05 ), *n* = 3.

cv. Maestà (Apulia Region)					
		As (µg/g d.m.) ± SE	Cd (µg/g d.m.) ± SE	Ni (µg/g d.m.) ± SE	Pb (µg/g d.m.) ± SE
Micronised sample		0.478 ± 0.037 ^a^	0.002 ± 0.0004 ^a^	0.043 ± 0.001 ^b^	0.157 ± 0.019 ^a^
Air-classified fractions	F 220	0.143 ± 0.004 ^d^	0.001 ± 0.0002 ^a^	0.031 ± 0.001 ^b^	0.059 ± 0.020 ^bc^
	F 230	0.190 ± 0.008 ^cd^	<LOD	0.027 ± 0.003 ^b^	0.030 ± 0.005 ^c^
	F 240	0.274 ± 0.012 ^bc^	0.001 ± 0.0002 ^a^	0.034 ± 0.002 ^b^	0.039 ± 0.004 ^c^
	F 250	0.359 ± 0.022 ^b^	<LOD	<LOD	0.005 ± 0.001 ^c^
	F 260	0.522 ± 0.016 ^a^	<LOD	0.355 ± 0.012 ^a^	0.038 ± 0.005 ^bc^
	F 270	0.511 ± 0.018 ^a^	0.074 ± 0.025 ^a^	0.058 ± 0.012 ^b^	0.103 ± 0.004 ^ab^
	F 280	0.513 ± 0.018 ^a^	0.001 ± 0.0003 ^a^	0.041 ± 0.013 ^b^	0.025 ± 0.006 ^c^
cv. Saragolla (Apulia Region)					
Micronised sample		0.518 ± 0.069 ^a^	0.008 ± 0.0013 ^abc^	0.143 ± 0.004 ^b^	0.152 ± 0.007 ^ab^
Air-classified fractions	F 220	0.073 ± 0.012 ^c^	0.001 ± 0.002 ^c^	0.014 ± 0.001 ^e^	0.074 ± 0.002 ^c^
	F 230	0.207 ± 0.009 ^bc^	0.005 ± 0.0004 ^bc^	0.039 ± 0.004 ^d^	0.077 ± 0.004 ^c^
	F 240	0.286 ± 0.014 ^b^	0.011 ± 0.003 ^a^	0.051 ± 0.002 ^cd^	0.081 ± 0.015 ^bc^
	F 250	0.486 ± 0.023 ^a^	0.005 ± 0.001 ^bc^	0.055 ± 0.002 ^c^	0.086 ± 0.011 ^bc^
	F 260	0.555 ± 0.029 ^a^	0.013 ± 0.003 ^ab^	0.057 ± 0.001 ^c^	0.163 ± 0.031 ^a^
	F 270	0.590 ± 0.038 ^a^	0.007 ± 0.002 ^abc^	0.126 ± 0.001 ^b^	0.118 ± 0.011 ^abc^
	F 280	0.297 ± 0.061 ^b^	<LOD	0.171 ± 0.003 ^a^	0.101 ± 0.026 ^abc^
cv. Iride (Apulia Region)					
Micronised sample		0.582 ± 0.013 ^a^	<LOD	<LOD	0.022 ± 0.005 ^b^
Air-classified fractions	F 220	0.110 ± 0.008 ^f^	0.001 ± 0.0002 ^b^	0.014 ± 0.001 ^d^	0.074 ± 0.002 ^d^
	F 230	0.246 ± 0.008 ^e^	<LOD	0.007 ± 0.002 ^d^	0.016 ± 0.001 ^d^
	F 240	0.342 ± 0.013 ^d^	<L.R.	0.009 ± 0.001 ^bcd^	0.038 ± 0.002 ^cd^
	F 250	0.469 ± 0.022 ^c^	<LOD	0.009 ± 0.001 ^cd^	0.038 ± 0.002 ^bc^
	F 260	0.502 ± 0.033 ^bc^	0.005 ± 0.0007 ^b^	0.031 ± 0.003 ^a^	0.055 ± 0.009 ^ab^
	F 270	0.618 ± 0.021 ^a^	<LOD	0.019 ± 0.003 ^bc^	0.044 ± 0.005 ^ab^
	F 280	0.638 ± 0.021 ^a^	0.028 ± 0.001 ^a^	0.021 ± 0.001 ^ab^	0.063 ± 0.006 ^a^

**Table 2:** Group I: average values ± standard error (SE) of arsenic (As), cadmium (Cd), nickel (Ni), and lead (Pb) contents in the micronised samples and G-type air-classified fractions; d.m.: dry matter; LOD: limit of detection , different letters indicate significant difference (*p* < 0.05 ), *n* = 3.

cv. Maestà (Apulia Region)					
		As (µg/g d.m.) ± SE	Cd (µg/g d.m.) ± SE	Ni (µg/g d.m.) ± SE	Pb (µg/g d.m.) ± SE
Micronised sample		0.478 ± 0.037 ^a^	0.002 ± 0.000 ^a^	0.043 ± 0.001 ^b^	0.157 ± 0.019 ^a^
Air-classified fractions	G 220	0.325 ± 0.034 ^b^	0.001 ± 0.000 ^ab^	0.102 ± 0.024 ^a^	0.109 ± 0.034 ^ab^
	G 230	0.313± 0.009 ^b^	0.002 ± 0.000 ^ab^	0.019 ± 0.001 ^c^	0.049 ± 0.0059 ^bc^
	G 240	0.372 ± 0.003 ^b^	<LOD	0.019 ± 0.002 ^c^	0.026 ± 0.003 ^c^
	G 250	0.145 ± 0.002 ^c^	0.001 ± 0.000 ^b^	0.001 ± 0.000 ^d^	0.006 ± 0.002 ^c^
	G 260	0.055 ± 0.000 ^d^	<LOD	0.003 ± 0.001 ^d^	0.003 ± 0.000 ^c^
	G 270	0.037 ± 0.001 ^d^	0.001 ± 0.000 ^ab^	0.002 ± 0.001 ^d^	0.007 ± 0.001 ^c^
	G 280	0.025 ± 0.001 ^d^	0.0001 ± 0.000 ^b^	0.003 ± 0.000 ^d^	0.007 ± 0.001 ^c^
cv. Saragolla (Apulia Region)					
Micronised sample		0.518 ± 0.069 ^a^	0.008 ± 0.0013 ^b^	0.143 ± 0.004 ^a^	0.152 ± 0.007 ^a^
Air-classified fractions	G 220	0.420 ± 0.044 ^ab^	0.021 ± 0.0056 ^a^	0.014 ± 0.001 ^bc^	0.213 ± 0.044 ^a^
	G 230	0.363 ± 0.001 ^bc^	<LOD	0.058 ± 0.0017 ^b^	0.061 ± 0.001 ^b^
	G 240	0.243 ± 0.005 ^cd^	0.001 ± 0.000 ^c^	0.008 ± 0.001 ^c^	0.036 ± 0.005 ^b^
	G 250	0.119 ± 0.002 ^de^	<LOD	0.007 ± 0.001 ^c^	0.015 ± 0.002 ^b^
	G 260	0.100 ± 0.002 ^de^	0.0007 ± 0.0001 ^c^	0.057 ± 0.001 ^bc^	0.020 ± 0.002 ^b^
	G 270	0.045 ± 0.001 ^e^	0.0005 ± 0.0001 ^c^	0.009 ± 0.003 ^c^	0.019 ± 0.001 ^b^
	G 280	0.034 ± 0.001 ^e^	0.0002 ± 0.000 ^c^	0.005 ± 0.001 ^c^	0.010 ± 0.001 ^b^
cv. Iride (Apulia Region)					
Micronised sample		0.582 ± 0.013 ^a^	<LOD	<LOD	0.022 ± 0.005 ^b^
air-classified fractions	G 220	0.455 ± 0.038 ^b^	<LOD	<LOD	0.044 ± 0.003 ^a^
	G 230	0.412± 0.002 ^b^	<LOD	<LOD	0.023 ± 0.0012 ^b^
	G 240	0.322 ± 0.002 ^c^	<LOD	<LOD	0.022 ± 0.002 ^b^
	G 250	0.160 ± 0.001 ^d^	0.008 ± 0.000 ^a^	0.035 ± 0.010 ^a^	0.015 ± 0.001 ^bc^
	G 260	0.088 ± 0.002 ^de^	<LOD	0.004 ± 0.001 ^b^	0.015 ± 0.002 ^bc^
	G 270	0.065 ± 0.001 ^e^	<LOD	<LOD	0.015 ± 0.001 ^bc^
	G 280	0.050 ± 0.001 ^e^	0.0003 ± 0.000 ^b^	<LOD	0.008 ± 0.001 ^c^

**Table 3:** Group II: average values ± standard error (SE) of arsenic (As), cadmium (Cd), nickel (Ni), and lead (Pb) contents in the micronised, semolina, and selected air-classified fractions (F250, G250, and G230); d.m.: dry matter; LOD: limit of detection, different letters indicate significant difference (*p* < 0.05 ), *n* = 3.

		As (µg/g d.m.) ± SE	Cd (µg/g d.m.) ± SE	Ni (µg/g d.m.) ± SE	Pb (µg/g d.m.) ± SE
cv. Saragolla (Lazio Region)	Micronised sample	0.100 ± 0.002 ^a^	0.01 ± 0.002 ^b^	<LOD	<LOD
	Semolina	0.053 ± 0.002 ^c^	0.002 ± 0.000 ^b^	<LOD	0.013 ± 0.001 ^a^
	F250	0.052 ± 0.004 ^c^	<LOD	<LOD	<LOD
	G250	0.025 ± 0.001 ^d^	0.01 ± 0.000 ^a^	<LOD	<LOD
	G230	0.063 ± 0.005 ^b^	0.002 ± 0.001 ^b^	<LOD	0.008 ± 0.003 ^a^
cv. Antalis (Basilicata Region)	Micronised sample	0.111 ± 0.05 ^a^	0.01 ± 0.001 ^a^	<LOD	0.138 ± 0.006 ^a^
	Semolina	0.066 ± 0.005 ^b^	0.001 ± 0.000 ^a^	<LOD	0.144 ± 0.026 ^a^
	F250	0.039 ± 0.001 ^c^	0.001 ± 0.000 ^a^	<LOD	0.042 ± 0.009 ^b^
	G250	0.015 ± 0.001 ^d^	0.001 ± 0.000 ^a^	<LOD	0.016 ± 0.002 ^c^
	G230	0.062 ± 0.007 ^b^	0.002 ± 0.000 ^a^	<LOD	0.038 ± 0.004 ^b^
cv. Antalis (Marche Region)	Micronised sample	0.164 ± 0.016 ^a^	<LOD	<LOD	<LOD
	Semolina	0.089 ± 0.007 ^b^	<LOD	<LOD	0.042 ± 0.002
	F250	0.041 ± 0.001 ^d^	<LOD	<LOD	<LOD
	G250	0.034 ± 0.002 ^e^	<LOD	<LOD	<LOD
	G230	0.054 ± 0.004 ^c^	<LOD	<LOD	<LOD

**Table 4:** Pasta: average values ± standard error (SE) of arsenic (As) content in semolina and selected air-classified fractions (F250, G250, and G230); d.m.: dry matter, different letters indicate significant difference (*p* < 0.05 ), *n* = 3.

As (µg/g d.m.) ± SE			
	**cv. Saragolla (Lazio Region)**	cv. Antalis (Basilicata Region)	cv. Antalis (Marche Region)
Semolina	0.050 ± 0.012 ^ab^	0.063 ± 0.009 ^ab^	0.048 ± 0.011 ^a^
F250	0.026 ± 0.004 ^b^	0.084 ± 0.019 ^a^	0.037 ± 0.004 ^a^
G250	0.023 ± 0.004 ^b^	0.031 ± 0.006 ^ab^	0.044 ± 0.010 ^a^
G230	0.053 ± 0.003 ^a^	0.029 ± 0.013 ^b^	0.039 ± 0.013 ^a^

## Data Availability

All data are contained within the article.
